# Supplementary material for: Synthesis of Bioactive Nickel Nanoparticles Using Bacterial Strains from an Antarctic Consortium
Source: Mar Drugs. 2024 Feb 14;22(2):89. doi: 10.3390/md22020089 (PMC10890439; doi:10.3390/md22020089)
Supplement: Supplementary file 1 [file marinedrugs-22-00089-s001.zip › marinedrugs-2868263-supplementary.pdf]

Supplementary materials

Table S1: Characteristics of NiSNPs synthesized by the different Antarctic bacteria

Characteristics	*Marinomonas*	*Pseudomonas*	*Rhodococcus*	*Brevundimonas*	*Bacillus*
Visual observation	Deposition of the pale green extracellular aggregates	Deposition of the pale green extracellular aggregates	Deposition of the pale green extracellular aggregates	Deposition of the pale green extracellular aggregates	Deposition of the pale green extracellula r aggregates
Synthesis time	24hrs	24hrs	24hrs	24hrs	24hrs
Uv spectroscopy	421nm	420nm	422nm	421nm	400nm
Dynamic Light Scattering	42.3 nm	42.1nm	44.8nm	40.7nm	40.5nm
Zeta potential measurement s	− 32.2 mV	− 28.5mV	− 31.1 mV	− 30.6 mV	− 29.3 mV
Transmission electron microscopy (size range)	Rods and spherical Spherical - 20-30nm Rods - 40-50nm	Rods and spherical Spherical - 20-30nm Rods -30-50nm	Rods and spherical Spherical - 15-30nm Rods of 25-50nm	Rods and spherical Spherical -20-30nm Rod - 40-50nm	Rods and spherical Spherical - 20-30nm Rods -30-50nm
X-ray powder diffraction analysis	Crystalline formation of a Ni monosulfide phase	Crystalline formation of a Ni monosulfide phase	Crystalline formation of a Ni monosulfide phase	Crystalline formation of a Ni monosulfide phase	Crystalline formation of a Ni monosulfid e phase

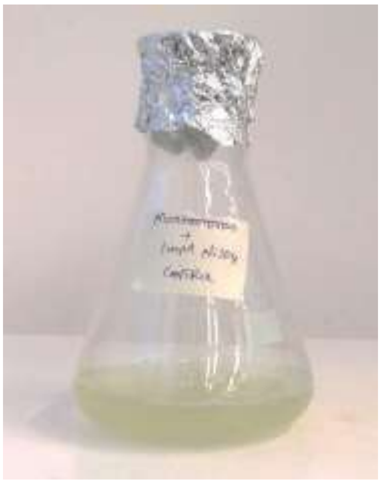
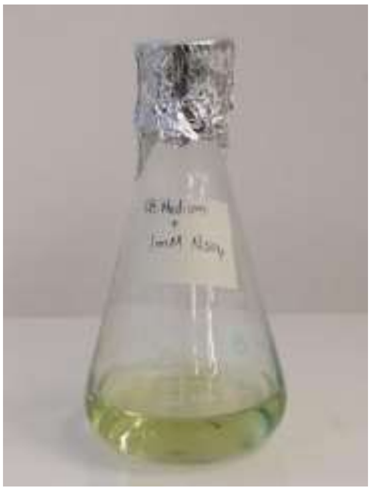
LB Medium
+
1mM NiSO4
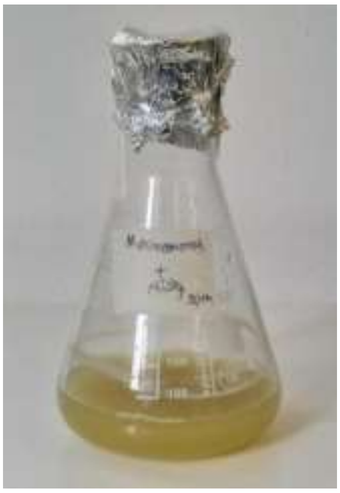
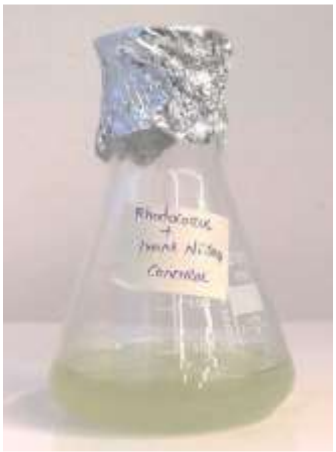
Rhodococcus
+
1mM NiSO4
Control
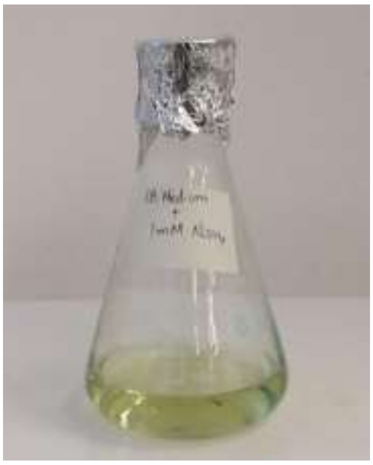
LB Medium
+
1mM NiSO4
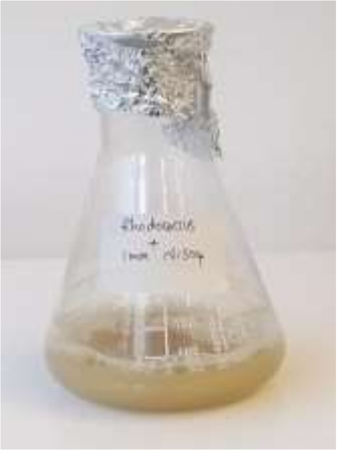
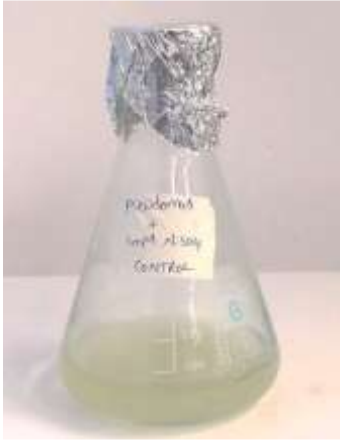
Control
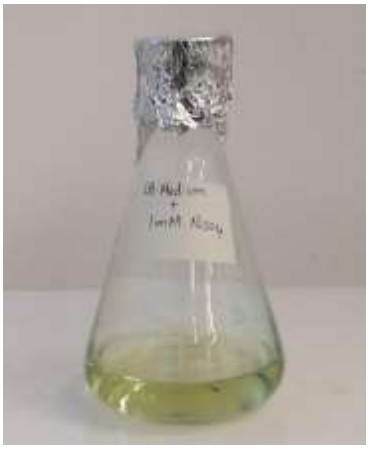
LB Medium
+
1mM NiSO4
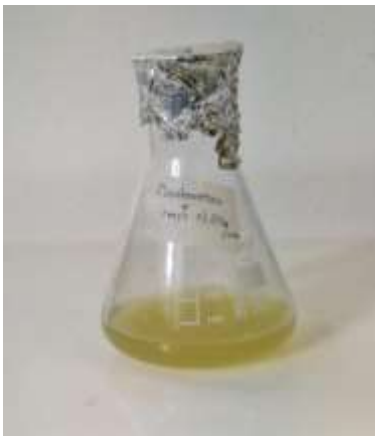

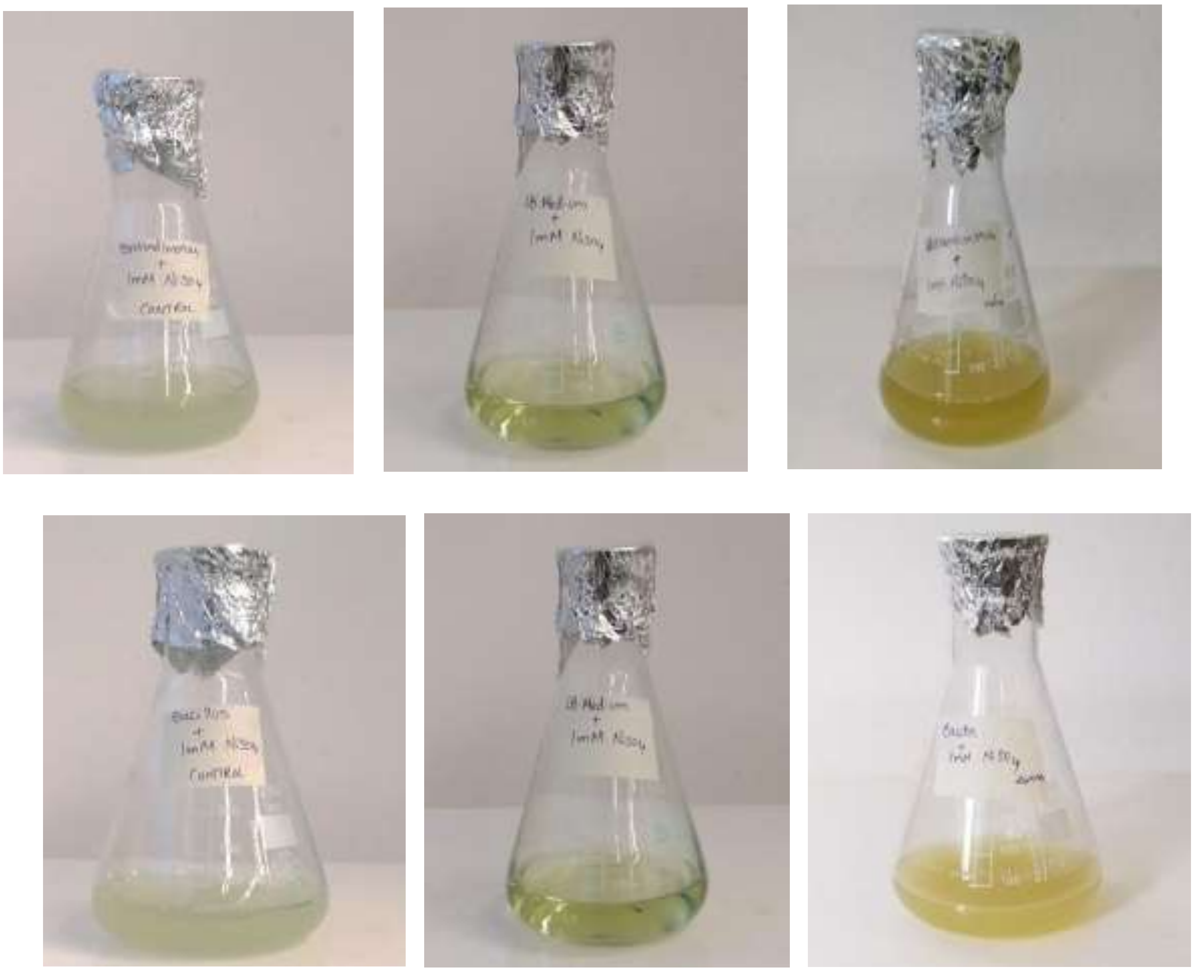

Figure S1: Biosynthesis of NiSNPs from *Marinomonas, Rhodococcus* ef1, *Brevundimonas* ef1, *Marinomonas* ef1, *Pseudomonas* ef1 *and Bacillus* ef1, respectively. Left panels: heat killed bacterial biomass with 1mM $NiSO_4$; Central panel: LB medium with 1mM $NiSO_4$; right panel: Biosynthesized NiSNPs

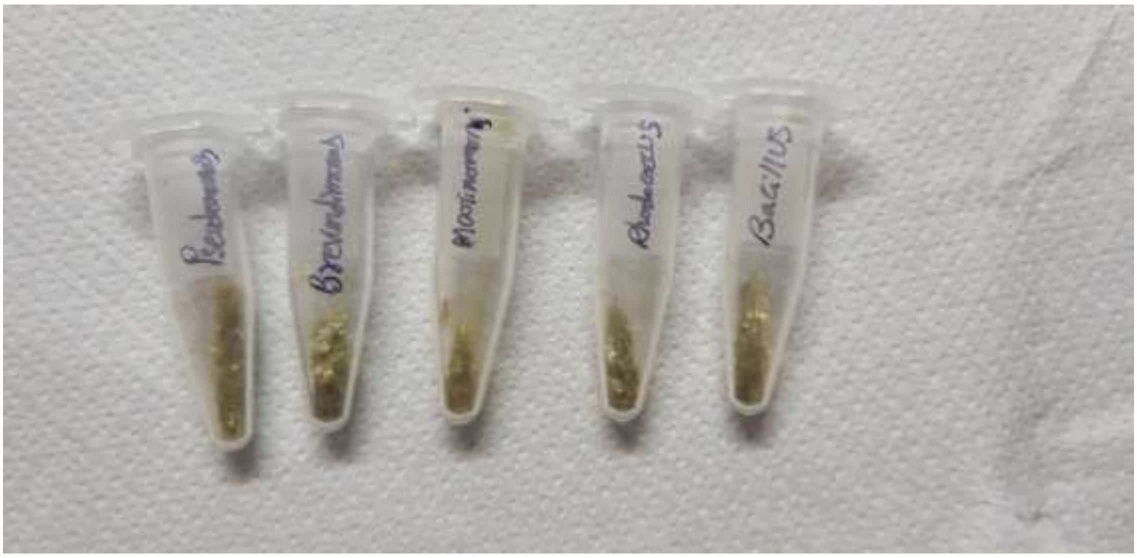

Figure S2. Purified NiSNPs from *Rhodococcus* ef1, *Brevundimonas* ef1, *Marinomonas* ef1, *Pseudomonas* ef1 *and Bacillus* ef1.

Table S2. Pathogens inhibition growth (expressed as diameter in mm) in the presence of bacterial NiSNPs and $NiSO_4$ (1mM), used as a control to verify the effect of nanoparticles and not of the salt.

	Marinomonas	*Rhodococcus*	*Brevundimonas*	*Pseudomonas*	*Bacillus*	$NiSO_4$
Staphylococcus aureus	14±0.2	15±0.2	14±0.3	14±0.1	15±0.1	11±0.2
Escherichia coli	14±0.2	14±0.2	13±0.3	14±0.2	13±0.4	9±0.2
Klebsiella pneumoniae	14±0.1	12±0.2	15±0.4	12±0.3	12±0.3	9±0.4
Pseudomonas sp	15±0.2	15±0.3	13±0.3	15±0.1	16±0.2	11±0.2
Proteus mirabilis	14±0.3	15±0.4	13±0.2	13±0.2	12±0.3	9±0.1
Citrobacter koseri	14±0.2	13±0.4	13±0.2	13±0.3	14±0.2	9±0.4
Acinetobacter baumanii	15±0.1	14±0.1	12±0.2	13±0.4	13±0.1	11±0.4
Serratia marcescens	14±0.2	12±0.2	13±0.1	13±0.2	13±0.2	8±0.3
Candida albicans	16±0.2	16±0.3	13±0.4	15±0.2	14±0.2	11±0.4
Candida parapsilosis	15±0.1	15±0.2	13±0.2	14±0.3	15±0.1	11±0.3

Supplementary results

1. FTIR spectroscopic analysis

***Marinomonas* ef1** showed the peak at 3252.83 cm^{-1} corresponds to O–H stretching carboxylic acids. Characteristic peaks at 2958.72 cm^{-1} indicate the possible presence of Aliphatic compounds CH_3 and $-CH_2$ stretch. The peak at 1560.76 cm^{-1} is attributed to N–H bend secondary amides. The sharp peak at 1402.29 cm^{-1} indicates C-N in primary amides. The peak at 1105.11^{-1} is primarily due to C–N stretching of aliphatic amines and the peak at 609.63 cm^{-1} is characteristic of NO_2 deformation in aliphatic nitro compounds.

FTIR spectrum of NiS NPs from *Marinomonas* ef1

A 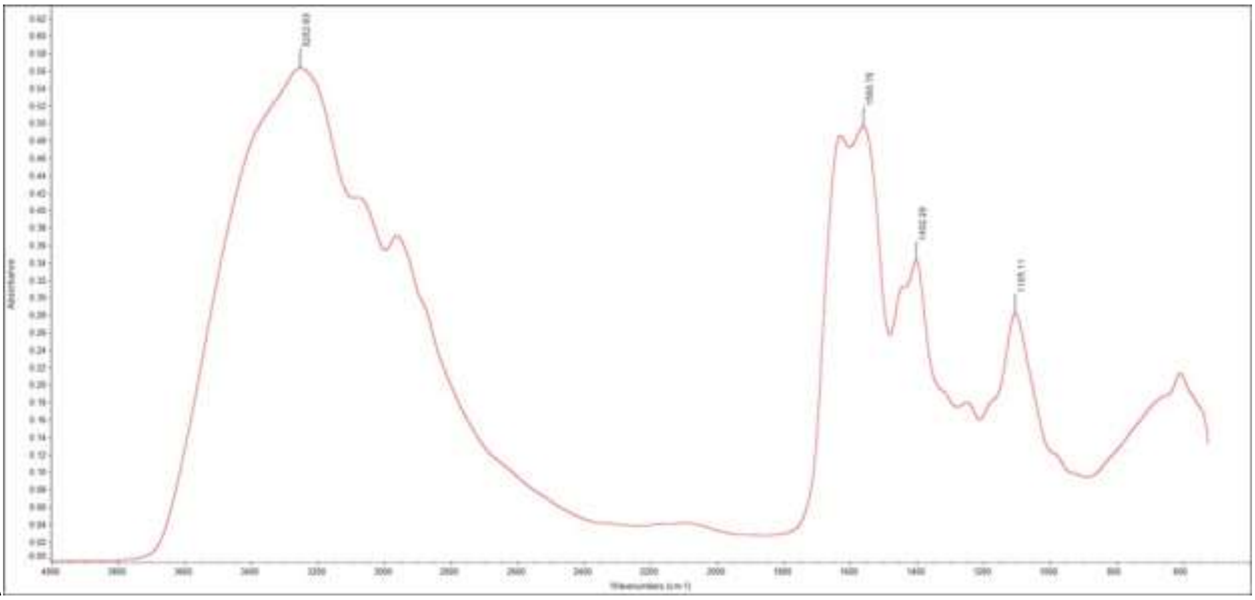

B

**Absorption cm^{-1}**	**Functional group**	**Characteristic Absorption(s)(cm^{-1})**
**3252.83**	**-OH group in alcohols and phenols**	**3260-3240**
**2958.72**	**CH_3 and $-CH_2$ stretch in Aliphatic compounds**	**2990-2850**
**1560.76**	**N-H stretch in secondary amides**	**1565-1475**
**1402.29**	**C-N stretch in primary amides**	**1420-1400**
**1105.11**	**C-C-N bending in amines**	**1230-1100**

***Rhodococcus* ef1** showed the peak at 3241.08cm^{-1} corresponds to O–H stretching carboxylic acids. Characteristic peak at 2959.96 cm^{-1} indicate the possible presence of Aliphatic compounds CH_3 and –CH2

2

stretch. The peak at 1566.58 cm^{-1} is attributed to NO in aliphatic nitro compounds. The sharp peak at 1399.16 cm^{-1} indicates COO- group in carboxylic acid salts. The peak at 1100.68cm^{-1} is primarily due to C–N stretching of aliphatic amines and the peak at 609.63 cm^{-1} is characteristic of NO deformation in aliphatic nitro compounds.

FTIR spectrum of NiS NPs from Rhodococcus

A

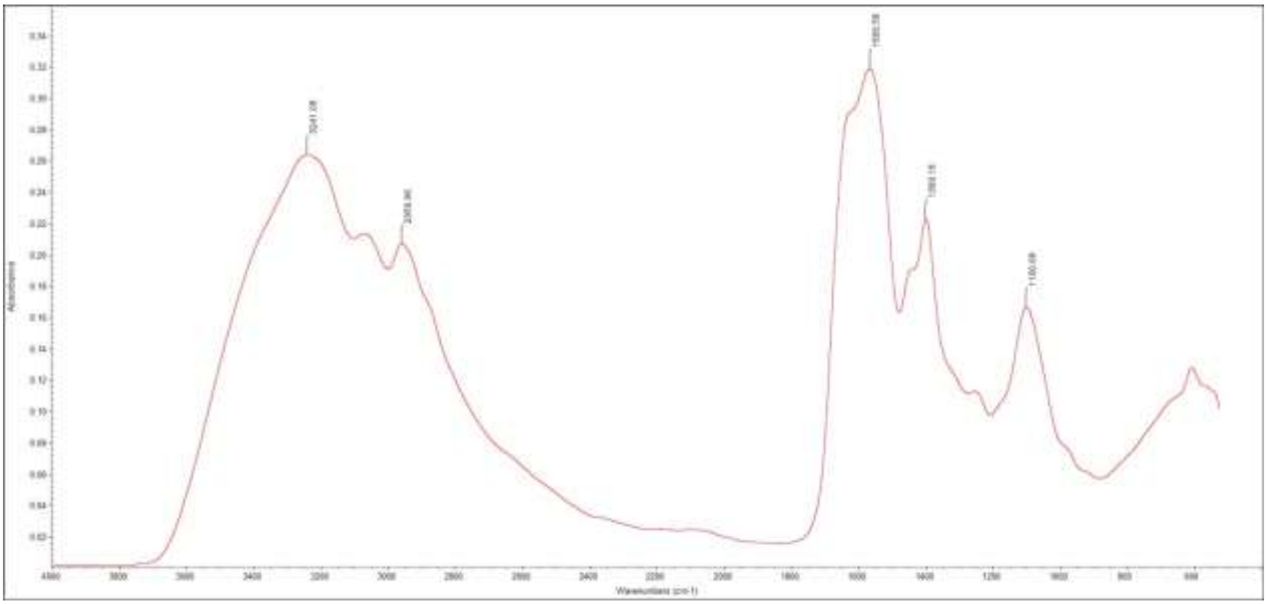

B

Absorption cm^{-1}	Functional group	Characteristic Absorption(s)(cm^{-1})
**3241.08**	**-OH group in alcohols and phenols**	**3260-3240**
**2959.96**	**CH_3 and –CH_2 stretch in Aliphatic compounds**	**2990-2850**
**1566.58**	**NO_2 stretch in aliphatic nitro compounds**	**1575-1545**
**1399.16**	**COO- group in carboxylic acid salts**	**1400-1310**
**1100.68**	**C-C-N bending in amines**	**1230-1100**

***Pseudomonas* ef1** showed the peak at 3259.31 cm^{-1} corresponds to O–H stretching carboxylic acids.

Characteristic peak at 2954.43 cm^{-1} indicates the possible presence of Aliphatic compounds CH and –CH stretch. The peak at 1576.72 cm^{-1} is attributed to NO in aliphatic nitro compounds. The sharp peak at 1399.63 cm^{-1} indicates COO- group in carboxylic acid salts. The peak at 1104.24 cm^{-1} is primarily due to C–N stretching of aliphatic amines and the peak at 609.63 cm^{-1} is characteristic of NO deformation in aliphatic nitro compounds.

FTIR spectrum of NiS NPs from *Pseudomonas* ef1

A

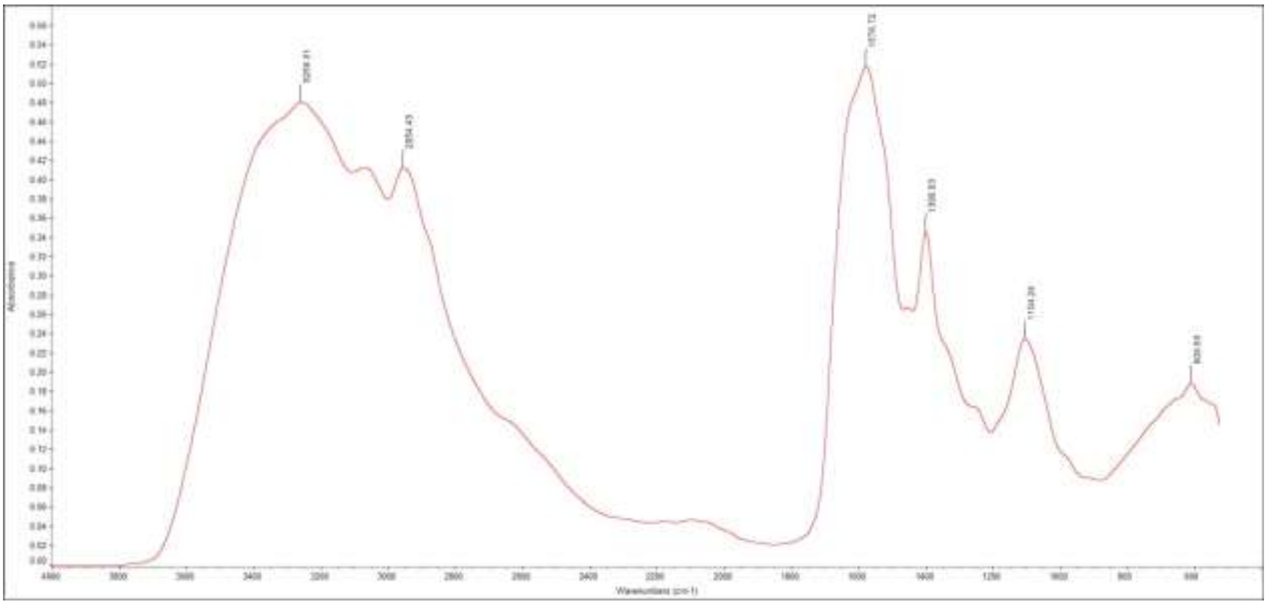

B

Absorption cm^{-1}	Functional group	Characteristic Absorption(s)(cm^{-1})
3259.31	-OH group in alcohols and phenols	3260-3240
2954.43	CH_3 and $–CH_2$ stretch in Aliphatic compounds	2990-2850
1576.72	COO^- group in carboxylic acid salts	1610-1560
1399.63	COO^- group in carboxylic acid salts	1400-1310
1104.24	C-C-N bending in amines	1230-1100
609.63	NO_2 deformation in aliphatic nitro compounds	650-600

Brevundimonas ef1 showed the peak at 3242.15 cm^{-1} corresponds to O–H stretching carboxylic acids. Characteristic peak at 2958.72 cm^{-1} indicate the possible presence of Aliphatic compounds CH_3 and $–CH_2$ stretch. The peaks at 1561.84 cm^{-1} and 1561.84 cm^{-1} are attributed to NO_2 in aliphatic nitro compounds and $-NH_2$ stretch of primary amines. The sharp peak at 1398.80 cm^{-1} indicates COO- group in carboxylic acid

salts. The peak at 1104.38 cm^{-1} is primarily due to C-C-N bending in amines and the peak at 608.30 cm^{-1} is characteristic of NO_2 deformation in aliphatic nitro compounds.

FTIR spectrum of NiS NPs from *Brevundimonas* ef1

A
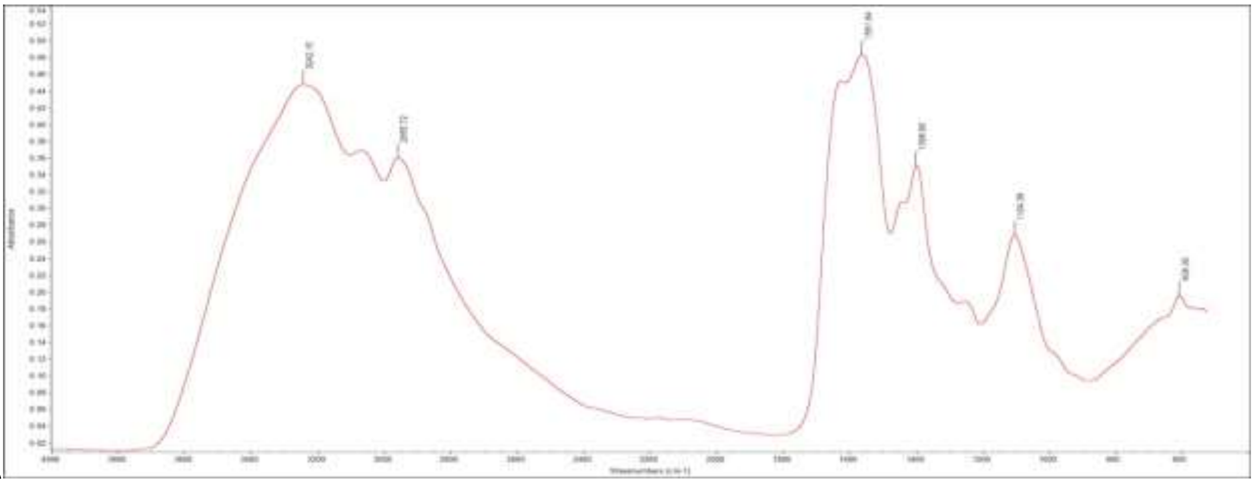

B

Absorption cm^{-1}	Functional group	Characteristic Absorption(s)(cm^{-1})
3242.15	-OH group in alcohols and phenols	3260-3240
2958.72	CH_3 and $–CH_2$ stretch in Aliphatic compounds	2990-2850
1561.84	NO_2 stretch in aliphatic nitrocompounds	1570-1550
1561.84	$-NH_2$ stretch of primary amines	1610-1580
1398.80	COO- group in carboxylic acid salts	1400-1310
1104.38	C-C-N bending in amines	1230-1100
608.30	NO_2 deformation in aliphatic nitro compounds	650-600

***Bacillus* ef1** showed the peak at 3242.15 cm^{-1} corresponds to O–H stretching carboxylic acids. Characteristic peak at 2958.72 cm^{-1} indicate the possible presence of Aliphatic compounds CH_3 and $–CH_2$ stretch. The peak at 1561.84 cm^{-1} is attributed to NO_2 in aliphatic nitro compounds. The sharp peak at 1398.80 cm^{-1} indicates COO- group in carboxylic acid salts. The peak at 1104.38 cm^{-1} is primarily due to C-C-N bending in amines and the peak at 608.30 cm^{-1} is characteristic of NO_2 deformation in aliphatic nitro compounds.

FTIR spectrum of NiS NPs from *Bacillus* ef1

A

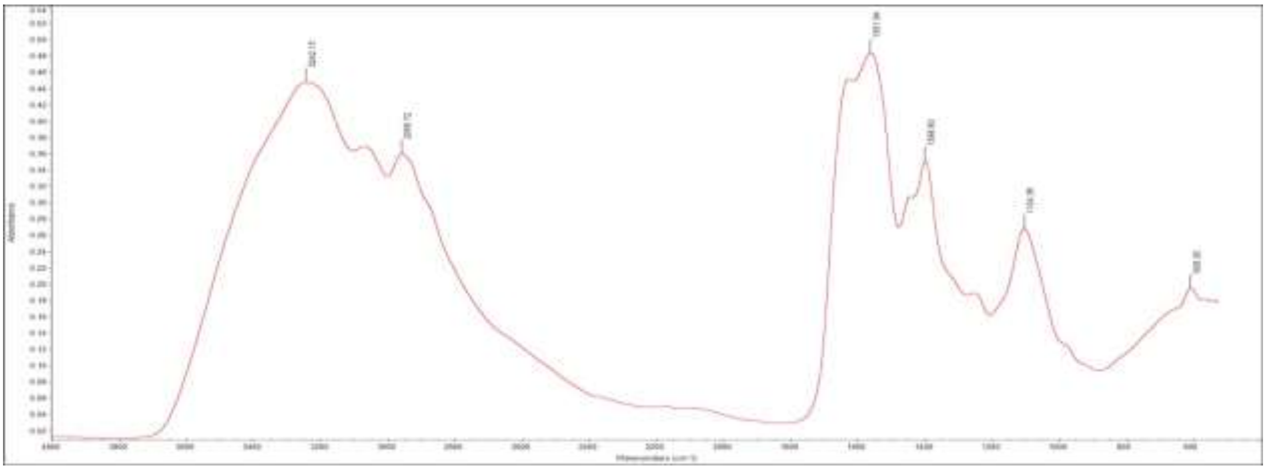

B

Absorption cm^{-1}	Functional group	Characteristic Absorption(s)(cm^{-1})
3242.15	-OH group in alcohols and phenols	3260-3240
2958.72	CH_3 and $-CH_2$ stretch in Aliphatic compounds	2990-2850
1561.84	NO_2 stretches in aliphatic nitro compounds	1570-1550
1398.80	COO- group in carboxylic acid salts	1400-1310
1104.38	C-C-N bending in amines	1230-1100

**608.30**	**NO_2 deformation in aliphatic nitro compounds**	**650-600**

2 MIC and MBC/MFC Assay

MIC values of ***Marinomonas* ef1** synthesized NiSNPs are shown in **Figure-S3**. Among the Gram-negative bacteria, *Proteus mirabilis* shows the lowest MIC of 3.12 µg/mL. *Citrobacter koseri* and *Serratia marcescens* show MIC values of 6.25 µg/mL. *Klebsiella pneumonia*, *Pseudomonas aeruginosa* and *Acinetobacter baumanii* show MIC values of 12.5 µg/mL. *Escherichia coli* shows a MIC value of 25 µg/mL. The gram positive bacterium *Staphylococcus aureus* shows a MIC value of 12.5 µg/mL. Among fungi, the lowest MIC value of 12.5 µg/mL is shown in *Candida parapsilosis*, while *Candida albicans* shows a MIC value of 25 µg/mL.

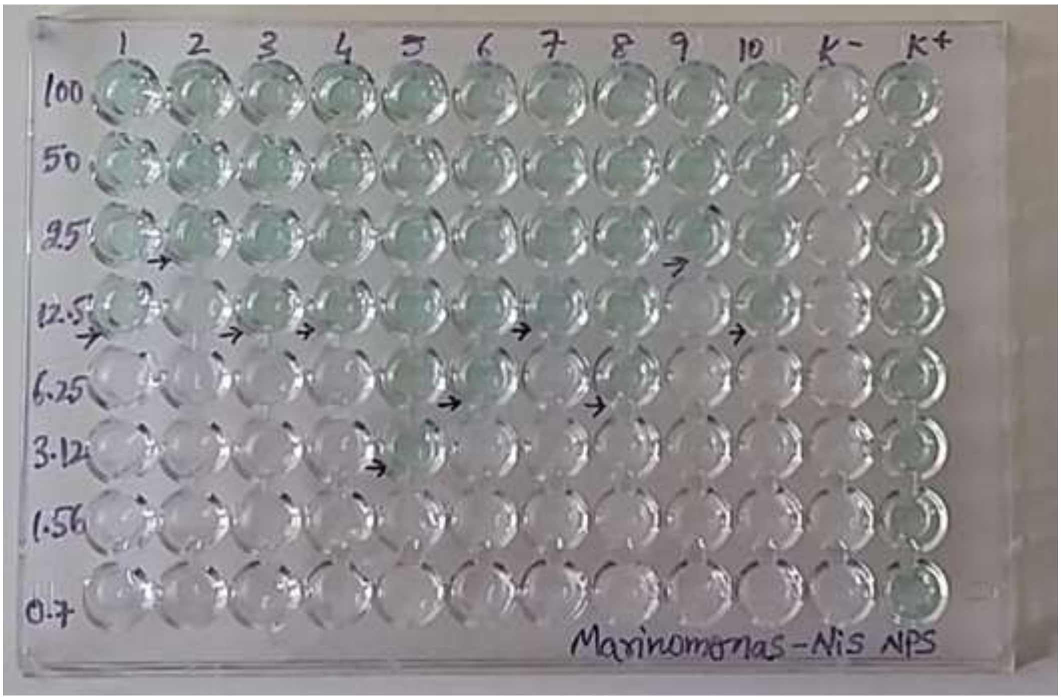

Figure-S3. MIC assay of the NiSNPs synthesized by *Marinomona*s ef1 by Broth microdilution method. 1. *Staphylococcus aureus* 2. *Escherichia coli* 3. *Klebsiella pneumoniae* 4. *Pseudomonas aeruginosa* 5. *Proteus mirabilis* 6. *Citrobacter koseri* 7. *Acinetobacter baumanii* 8. *Serratia marcescens* 9. *Candida albicans* 10. *Candida parapsilosis*. Positive control contains only medium (K+) and Negative control contains medium and bacterial inoculums (K-).

Among gram negative bacteria, *Proteus mirabilis* shows the lowest MBC value of 6.25 µg/mL, while *Pseudomonas aeruginosa*, *Citrobacter koser*i and *Acinetobacter baumanii* and *Serratia marcescens* show MBC values of 12.5 µg/mL. *Escherichia coli* and *Klebsiella pneumonia* show MBC values of 25 µg/mL. The gram positive bacterium *Staphylococcus aureus* shows a MBC value of 25 µg/mL. The Fungi *Candida albicans* and *Candida parapsilosis* show MFC values of 25 µg/mL.

MIC values of ***Rhodococcus*** synthesized NiSNPs are shown in **Figure-S4**. Among Gram negative bacteria, *Proteus mirabilis, Acinetobacter baumanii* and *Serratia marcescens* show the lowest MIC values of 6.25 µg/mL, whereas *Escherichia coli, Klebsiella pneumonia, Pseudomonas aeruginosa* and *Citrobacter koseri* show MIC values of 12.5 µg/mL. *Escherichia coli, Pseudomonas aeruginosa, Citrobacter koseri* and *Acinetobacter baumanii* show MIC values of 12.5 µg/mL. The gram positive bacteria *Staphylococcus aureus* shows a MIC value of 25 µg/mL. Among fungi, the lowest MIC value of 12.5 µg/mL is noted in *Candida albican*s, while *Candida parapsilosis* shows a MIC value of 25 µg/mL.

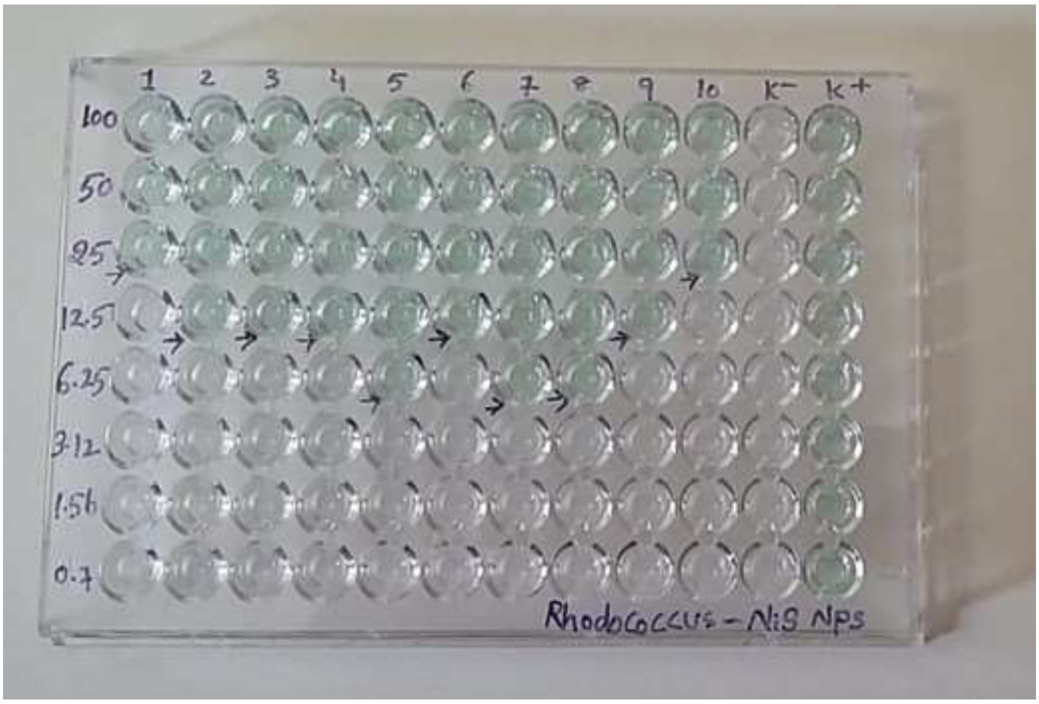

Figure-S4. MIC assay of the NiS NPs synthesized by *Rhodococcus* by Broth microdilution method. *1. Staphylococcus aureus 2. Escherichia coli 3. Klebsiella pneumoniae 4. Pseudomonas sp 5. Proteus mirabilis 6. Citrobacter koseri 7. Acinetobacter baumanii 8. Serratia marcescens 9. Candida albicans 10. Candida parapsilosis.*

The lowest MBC value among gram negative bacteria of 12.5 µg/mL is shown by *Proteus mirabilis, Citrobacter koseri, Acinetobacter baumanii* and *Serratia marcescens. Escherichia coli, Klebsiella pneumonia* and *Pseudomonas aeruginosa* showed MBC values of 25 µg/mL. The gram positive bacterium *Staphylococcus aureus* showed an MBC of 25 µg/mL. The Fungi C*andida albicans* and *Candida parapsilosis* also showed MFC values of 25 µg/mL.

MIC values of ***Pseudomonas* ef1** synthesized NiSNPs are shown in **Figure-S5**. Among Gram-negative bacteria, *Proteus mirabilis* and *Citrobacter koseri* show the lowest MIC value of 6.25 µg/mL, whereas *Pseudomonas aeruginosa, Acinetobacter baumanii a*nd *Serratia marcescens* show MIC values of 12.5 µg/mL. *Escherichia coli* and *Klebsiella pneumonia* show MIC values of 25 µg/mL. The gram positive bacterium *Staphylococcus aureus* shows a MIC value of 25µg/mL. Among fungi, *Candida parapsilosis* shows a MIC value of 12.5 µg/mL, while *Candida albicans* shows a MIC value of 25 µg/mL.

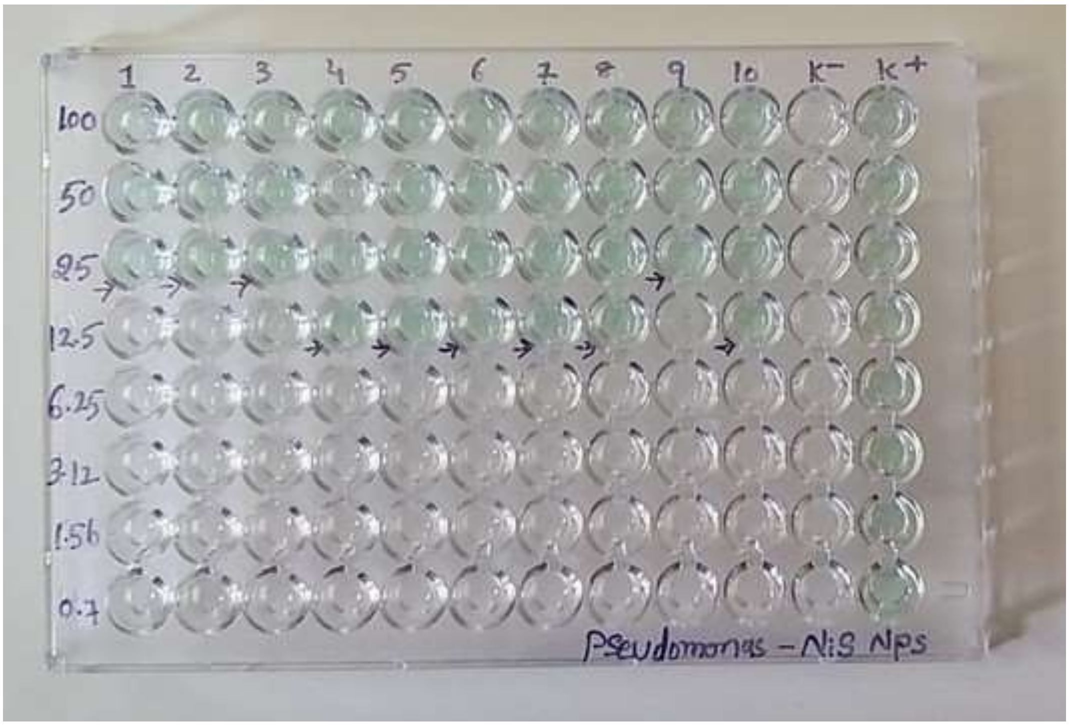

Figure-S5.MIC assay of the NiS NPs synthesized by *Pseudomonas* ef1 by Broth microdilution method. *1. Staphylococcus aureus 2. Escherichia coli 3. Klebsiella pneumoniae 4. Pseudomonas sp 5. Proteus mirabilis 6. Citrobacter koseri 7.*

Acinetobacter baumanii 8. Serratia marcescens 9. Candida albicans 10.

The lowest MBC value of 12.5 µg/mL among gram negative bacteria is shown by *Pseudomonas aeruginosa, Proteus mirabilis, Citrobacter koseri, Acinetobacter baumanii* and *Serratia marcescens*. *Escherichia coli* and *Klebsiella pneumonia* showed an MBC value of 25µg/mL. The gram positive bacterium *Staphylococcus aureus* showed an MBC of 25 µg/mL. The Fungi *Candida albicans* and *Candida parapsilosis* showed also a MFC value of 25 µg/mL.

MIC values of ***Brevundimonas*** synthesized NiSNPs are shown in **Figure-S6**. Among Gram-negative bacteria, *Serratia marcescens* shows the lowest MIC value of 6.25 µg/mL, whereas *Klebsiella pneumonia, Pseudomonas aeruginosa, Proteus mirabilis, Citrobacter koseri* and *Acinetobacter baumanii* show a MIC value of 12.5 µg/mL. *Escherichia coli* shows a MIC value of 25 µg/mL. The gram positive bacterium

Staphylococcus aureus shows a MIC value of 12.5 µg/mL. The fungi *Candida albicans* and *Candida parapsilosis* showed a MIC value of 12.5 µg/mL.

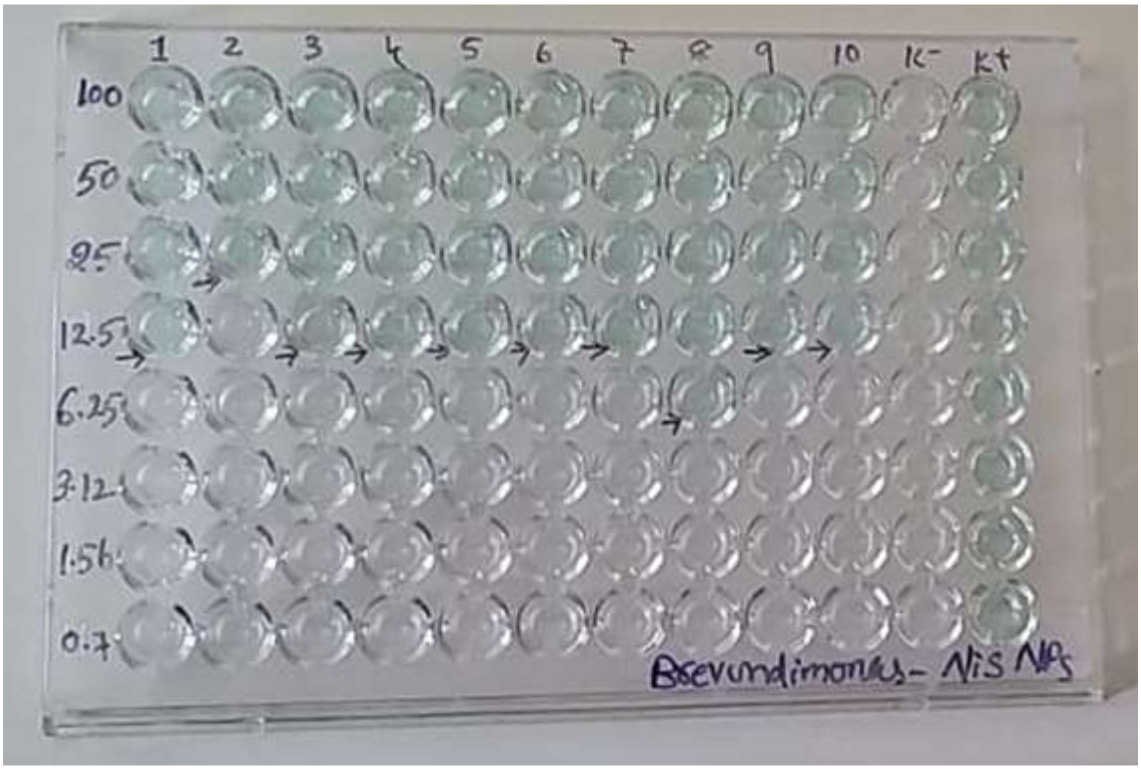

Figure S6. MIC assay of the NiS NPs synthesized by Brevundimonas by Broth microdilution method. ***1. Staphylococcus aureus 2. Escherichia coli 3. Klebsiella pneumoniae 4. Pseudomonas aeruginosa 5. Proteus mirabilis 6. Citrobacter koseri 7. Acinetobacter baumanii 8. Serratia marcescens 9. Candida albicans 10. Candida parapsilosis***

Among gram negative bacteria, the lowest MBC value of 12.5 µg/mL is shown by *Proteus mirabilis* and *Serratia marcescens*. *Escherichia coli, Klebsiella pneumonia, Pseudomonas aeruginosa, Citrobacter koseri and Acinetobacter bauman*ii show MBC values of 25 µg/mL. The gram-positive bacterium *Staphylococcus aureus* shows a MBC of 25 µg/mL. Also the Fungi *Candida albicans* and *Candida parapsilosis* shows MFC values of 25 µg/mL.

MIC values of ***Bacillus*** synthesized NiSNPs are shown in **Figure-S7**. Among Gram-negative bacteria, *Klebsiella pneumonia, Proteus mirabilis* and *Serratia marcescens* show the lowest MIC values of 6.25 µg/mL, whereas *Escherichia coli, Pseudomonas aeruginosa, Citrobacter koseri* and *Acinetobacter baumanii* show MIC values of 12.5 µg/mL. The gram-positive bacterium *Staphylococcus aureus* shows a MIC value of 12.5 µg/mL. Among fungi, *Candida parapsilosis* shows a MIC value of 6.25 µg/mL whereas *Candida albicans* of 12.5 µg/mL.

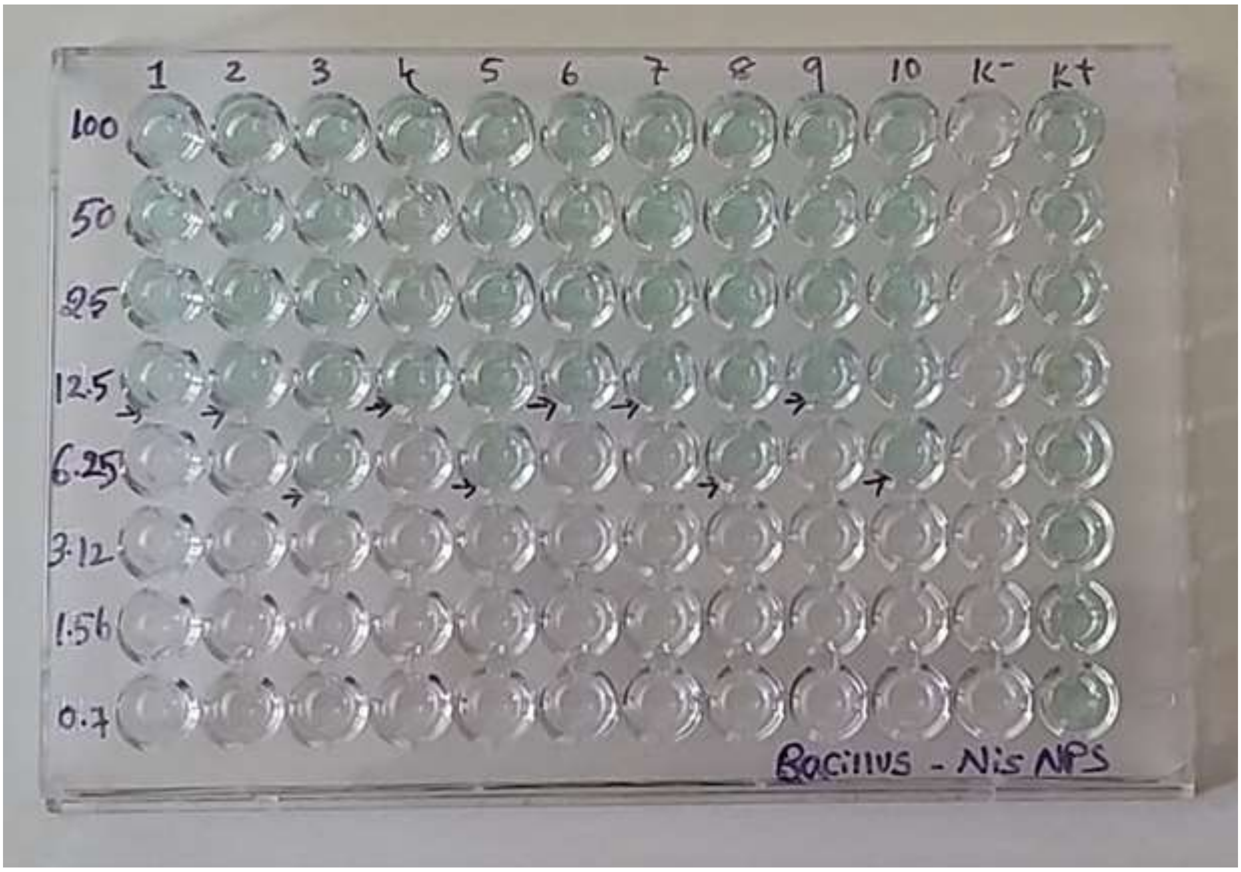

Figure-S7.MIC assay of the NiS NPs synthesized by Bacillus by Broth microdilution method.*1. Staphylococcus aureus 2. Escherichia coli 3. Klebsiella pneumoniae 4. Pseudomonas aeruginosa 5. Proteus mirabilis 6. Citrobacter koseri 7. Acinetobacter baumanii 8. Serratia marcescens 9. Candida albicans 10. Candida parapsilosis*

The lowest MBC value of 12.5µg/mL among the gram-negative bacteria is shown by *Escherichia coli, Klebsiella pneumonia, Pseudomonas aeruginosa, Proteus mirabilis, Citrobacter koseri, Acinetobacter baumanii* and *Serratia marcescens*. The gram-positive bacterium *Staphylococcus aureus* shows a MBC of 12.5 µg/mL. The Fungi *Candida albicans* and *Candida parapsilosis* show MFC values of 25 µg/mL and 12.5 µg/mL respectively.
